# Construction of a Highly Active Xylanase Displaying Oleaginous Yeast: Comparison of Anchoring Systems

**DOI:** 10.1371/journal.pone.0095128

**Published:** 2014-04-17

**Authors:** Sophie Duquesne, Sophie Bozonnet, Florence Bordes, Claire Dumon, Jean-Marc Nicaud, Alain Marty

**Affiliations:** 1 Université de Toulouse; INSA, UPS, INP; LISBP, Toulouse, France; 2 INRA, UMR792 Ingénierie des Systèmes Biologiques et des Procédés, Toulouse, France; 3 CNRS, UMR5504, Toulouse, France; 4 INRA, UMR1319 Micalis, Domaine de Vilvert, Jouy-en-Josas, France; 5 AgroParisTech, UMR Micalis, Jouy-en-Josas, France; University Paris South, France

## Abstract

Three *Yarrowia lipolytica* cell wall proteins (YlPir, YlCWP1 and YlCBM) were evaluated for their ability to display the xylanase TxXYN from *Thermobacillus xylanilyticus* on the cell surface of *Y. lipolytica*. The fusion proteins were produced in *Y. lipolytica* JMY1212, a strain engineered for mono-copy chromosomal insertion, and enabling accurate comparison of anchoring systems. The construction using YlPir enabled cell bound xylanase activity to be maximised (71.6 U/g). Although 48% of the activity was released in the supernatant, probably due to proteolysis at the fusion zone, this system is three times more efficient for the anchoring of TxXYN than the YlCWP1 system formerly developed for *Y. lipolytica*. As far as we know it represents the best displayed xylanase activity ever published. It could be an attractive alternative anchoring system to display enzymes in *Y. lipolytica*.

## Introduction

Nowadays, the use of enzymes as catalysts in various industrial transformations is widespread due to the relative ease of their manipulation and, when compared to chemical processes, their ability to perform reactions in eco-friendly conditions (i.e. in aqueous solutions at low temperatures). Nevertheless, using free enzymes in processes is often costly, since in this form the catalyst is not easily recycled. The conventional strategy to surmount this handicap is to operate continuous processes using immobilized enzymes. Many *in vitro* immobilisation techniques were developped, but enzymes have also been immobilized *in vivo* at the surface of the microorganisms, particularly yeast. This latter strategy is powerful because it eliminates the need for enzyme purification, provides a direct link between the enzyme's function and its gene and potentially couples enzyme action to other cellular metabolic processes, and thus opens the way to multi-step bioconversion processes [1].

*In vivo* displayed enzymes are usually covalently-linked to the cell wall of microorganisms, using three main strategies. The first of these employs a Glycosyl Phosphatidyl Inositol (GPI)-anchoring system, where the target enzyme is fused to a GPI-Cell Wall Protein (CWP), such as α-agglutinin, which provides the means to create a covalent bond between the fusion protein and cell wall β-1,6 glucans [2]. Another widely used display system, based on disulfide covalent linkage, is the fusion of the target enzyme to Aga2, an α-agglutinin subunit. This allows the fusion protein to form disulphide bonds with Aga1, another α-agglutinin subunit, which is in turn covalently linked to the cell wall through GPI anchor. The third system, called Protein Internal Repeat (or Pir)-CWP, is unrelated to the former and permits the covalent linkage of fusion proteins both to cell wall β-1,3 glucans and to structural proteins via disulfide bonds [3], [4]. Finally, an alternative, but rarely employed system consists of the non-covalent adsorption of an enzyme to the cell surface via interactions with a Chitin Binding Module (CBM) [5].

So far, most work performed on yeast cell surface protein display has targeted lipolytic enzymes [6], [7], or more occasionally amylolytic [8], [9] or cellulolytic/hemicellulolytic enzymes [10]–[12] and has generally involved the use of *Pichia* sp. or *Saccharomyces* sp. strains. In these yeasts, the success of protein display is dependent on the exact nature of the target protein and on the anchoring domain employed. Remarkably, despite this latter observation, no comprehensive comparison of the different anchoring systems (i.e. GPI, glucan ester, disulphide bonds and non-covalent) has yet been performed.

The main aim of the present study was to compare the performance of covalent and non-covalent cell surface anchoring systems in yeast, using a single target enzyme (and thus enzyme-coding sequence) and a system that provides the means to control the copy number and the genetic insertion locus of the fusion encoding sequences, thus maximizing the probability of obtaining constant expression levels irrespective of the construction. This last point is important because a recent study performed by Yuzbasheva *et al.*, which compared the performance of different C-domains of 6 GPI-anchored cell wall proteins as tools for the display of the lipase Lip2 on the surface of wild type *Yarrowia lipolytica* using random gene integration, revealed a high variance coefficient between several transformants of a single construction [13].

To achieve the goal of our study, we have employed the *Y. lipolytica* strain JMY1212 [14]. In previous work, this strain was shown to be a suitable host for the expression of secreted recombinant proteins, allowing post-translational modifications, including glycosylation, but without the disadvantage of hyper-glycosylation [15]. Moreover, it has been shown that the use of *Y. lipolytica* JMY1212 permits the directed insertion of expression cassettes into a specific docking platform [16]. This latter characteristic makes *Y. lipolytica* JMY1212 ideal for the rapid and convenient comparison of secreted enzyme variants, drastically reducing the variability that is inherent to the introduction of gene cassettes into the wild type strain. Finally, it is noteworthy that *Y. lipolytica* is a GRAS (generally regarded as safe) microorganism, thus the study of enzyme display in this microorganism is particularly interesting from an industrial perspective, especially since our study extends the investigation of enzyme display in *Y. lipolytica* beyond the use of CWP1 and homologues as anchoring proteins [13], [17].

The second aim of this work was to express a biomass-degrading enzyme at the surface of *Y. lipolytica*. For this, the CAZy family GH11 xylanase from *Thermobacillus xylanilyticus*, TxXYN, was chosen as target enzyme. Xylanases are among the key polysaccharide-degrading enzymes that are necessary to hydrolyse industrially-relevant lignocellulosic biomass in biorefinery processes [18], and are also already widespread in industry, being used in paper pulping and feed and food processing [19]. Regarding the choice of TxXYN, this enzyme is a well-characterized thermostable, highly active xylanase that has previously been submitted to protein engineering [20].

## Materials and Methods

### 1. Chemicals

Tryptone and yeast nitrogen base (without amino acids and without ammonium sulphate) were purchased from Difco (Difco, Paris, France). Kanamycin (used at 40 µg/mL) was purchased from Euromedex (Souffelweyersheim, France). Unless otherwise stated, chemicals were purchased from Sigma-Aldrich (Sigma-Aldrich, St. Louis, Mo.).

### 2. Strains and plasmids

The plasmid construction (pECXYL-R2) containing the coding sequence of the mature enzyme (Genbank accession AM237841.1) was described elsewhere [20]. *E. coli* strain JM109 (DE3) (genotype: *end*A1, *rec*A1, *gyr*A96, *thi, hsd*R17 (r_k_^−^, m_k_^+^), *rel*A1, *sup*E44, λ–, Δ(*lac-pro*AB), [F′, *tra*D36, *pro*AB, *lac*I^q^ZΔM15], lDE3), transformed with this plasmid was used to produce the free xylanase.

Chemically competent *E. coli* DH5α (Φ80dlacZΔm15, recA1, endA1, gyrA96, thi-1, hsdR17 (rk−, mk+), supE44, relA1, deoR, Δ(lacZYAargF) U169) were purchased from Invitrogen.

The strain *Y. lipolytica* JMY1212 (*MATA ura3*-*302 leu*2-*270*-*LEU2*-zeta *xpr2-322 Δlip2 Δlip7 Δlip8*, Leu+, Ura−) was used as expression system for both the free xylanase and the anchored xylanase.

The JMP62-TEF-*ppLIP2*-*LIP2* expression vector used for the construction of other expression plasmids is a derivative of the expression vector described in [15]. It contains the *ura3d1* marker for selection of Ura+ transformants in *Y. lipolytica* and the kanamycin gene (*Kan*R) for selection in *E. coli*. The gene of interest is under the control of the constitutive TEF promoter.

### 3. Construction of fusion proteins

Protein and nucleotide sequences refer to UniProtKB accession number (http://www.uniprot.org/uniprot/) and Génolevures database (http://genolevures.org/); N-glycosylation sites were predicted with http://www.cbs.dtu.dk/services/NetNGlyc/.

Genomic DNA from *Y. lipolytica* was prepared as already described [21]. PCR amplifications were performed on a MyCycler ThermalCycler from Biorad using Phusion polymerase from New England Biolabs (Beverly, MA, USA) using specific primer pairs that are described in table 1.

**Table 1:** Oligonucleotides used in this study to amplify gene fragments.

Oligonucleotide Reference Number	Restriction site	5′-3′ Sequence
1		GAGGCCGCAGTTCTCCAGAAGCGAAACACGTACTGGCAG
2	*Avr* II	CCTAGG TTACCAAACCGTGACGTTGGAGTATCC
3	*Hin* dIII	GCCATG AAGCTT TCCACCATCCTTTTCACAGCCTGCGCTAC
4		GCCATGAAGCTTTCCACCATCCTTTTCACAGCCTGCGCTAC
5	*Bgl* I, *Hin*dIII	CCGGCG AGATCT CACAATGAAGCTTTCCACCATCC
6	*Avr* II	CCTAGG CCGCGGGGATCCGGCGGCAGCCAGGGTAGCG
7	*Avr* II	GGATCCCCGCGG CCTAGGGGCTCCTCCGGAGGTGGTTCCGGCTCCGGC ***CACCACCACCACCACCAC*** GCTAGGGGCAACGGTTACGCCGTCG
8	*Nhe* I	GGCCT GCTAGC TTAAATGAGGAGAGCGGCGGC
9	*Hin*dIII	CGCCATGAAGCTTTCCACCATCC
10	*Avr* II	CAGACAC CCTAGG TGACCAAACC
11	*Bsi*WI	GACGCCGTACGCCGCTACTTCTTCCGCTGCTTCTTCTGC
12	*Bsr*GI	GCGTCTGTACAC ***GTGGTGGTGGTGGTGGTG*** GGCGTCGATATAGTTGCAGCCGACC
13	*Bsi*WI	GACGCCGTACGCTTGCAAGAAGGAGGGTACTCTCGCC
14	*Bsr*GI	GCGTCTGTACAC ***GTGGTGGTGGTGGTGGTG*** ACAGTCCTCGAGGTTGACAATGGCG
15	*Bsr*GI	GACGCTGTACAACACGTACTGGCAGTATTGGACGG
16	*Avr* II	GCTTAGATACCACAGACAC CCTAGGTTA

Restriction sites used for the construction are listed and underlined in the sequences. To allow western blot analyses, a nucleic acid sequence encoding six histidines was inserted between the sequences of TxXYN and anchoring protein domains (italic bold).

For the construction of JMP61-POX2-*ppLIP2*-*TxXYN*, the sequence of the *LIP2* prepropeptide (*ppLIP2*), which directs the secretion of recombinant proteins, was amplified by PCR from JMP8 vector [14], using primers 3 and 4. Similarly, the sequence encoding TxXYN was amplified from pECXYL-R2 vector using primers 1 and 2. Primer 2 contains a 16-base pair 5′ tail that overlaps with the *ppLIP2*-encoding sequence. Finally, to assemble the fusion protein *ppLIP2*-*Tx*XYN a fusion PCR was performed using primers 1 and 4. After purification (QIAquick PCR purification kit, QIAgen, Venlo, Netherlands), the amplified DNA was digested by *Hin*dIII and *Avr*II, and ligated to similarly digested JMP61, thus procuring JMP61-*ppLIP2*-*TxXYN*. In this construction, the xylanase gene is placed under the control of the POX2 promoter, whose activity is induced in the presence of oleic acid.

To construct JMP62-TEF-*ppLIP2-Pir100-TxXYN* and JMP62-TEF-*ppLIP2-CBM87-TxXYN*, anchoring proteins CBM87 and Pir100 were amplified by PCR using JMY1212 genomic DNA as the template and primers 11/12 and 13/14 respectively. The use of these primers directed the introduction of *Bsi*WI and *Bsr*GI restriction sites into the amplified DNA fragments, and since *Bsi*WI and *Bsr*GI generate compatible extremities, the subsequent ligation of the fragments to *Bsr*GI-digested JMP62-TEF-*ppLIP2-LIP2* re-introduced a *Bsr*GI site downstream of *Pir100* and *CBM87*, while annihilating the upstream site. The replacement of *LIP2* by *TxXYN* in the resulting plasmids JMP62-TEF-*ppLIP2-Pir100-LIP2* and JMP62-TEF-*ppLIP2-CBM87-LIP2* was obtained by double digestion of the plasmid construction and insertion of the amplified *TxXYN* coding sequence from plasmid JMP61-*ppLIP2*-*TxXYN* (primers 15/16 -flanking ends *Bsr*GI/*Avr*II) between *Bsr*GI and *Avr*II.

The construction JMP62-TEF-*ppLIP2-TxXYN*-*CWP110* was prepared first by amplifying the sequence encoding the C-terminal 110 amino acids of CWP1 (*CWP110*) using JMY1212 genomic DNA as template and primers 7 and 8, which introduced sites for *Avr*II and *Nhe*I respectively. Next, JMP62-TEF-*ppLIP2-TxXYN-CWP110* was constructed by fusing the sequence encoding *preLIP2*, obtained by PCR amplification using JMP62-TEF-*ppLIP2-LIP2* as the template and primer 5 and 6 (these introduced sites for *Bgl*I and *Avr*II respectively) to the sequence encoding *CWP110*. This sequence fusion, designed to create a new *Avr*II restriction site between *preLIP2* and *CWP110*, was then inserted into the vector JMP62-TEF-*ppLIP2*-*LIP2* between *Bam*HI and *Avr*II (compatible ends with *Bgl*I and *Nhe*I respectively), resulting in plasmid JMP62-TEF-*preLIP2-CWP110*. It is noteworthy that the construction of this fusion sequence removed flanking *Bam*HI, *Avr*II, *Bgl*I and *Nhe*I restriction sites, leaving only the internal *Avr*II restriction sites available for further cloning work. Afterwards, *ppLIP2*-*TxXYN* was then inserted into JMP62-TEF-*preLIP2-CWP110* between *Hin*dIII and *Avr*II sites. This was achieved by amplifying the *ppLIP2*-*TxXYN* encoding sequence using the JMP61-*ppLIP2*-*TxXYN* as the template and primers 9 and 10, which introduced *Hin*dIII and *Avr*II at the 5′ and 3′ extremities of the PCR fragment respectively, followed by restriction digestion and ligation. In the resulting construction, the stop codon present in the original *TxXYN* encoding sequence was replaced by a serine, thus facilitating fusion to *CWP110* at its C-terminal. In the three latter constructions, the xylanase encoding sequence was placed under the control of TEF constitutive promoter.

After transformation in *E. coli*, all of the plasmid constructions were verified using DNA sequencing (GATC Biotech, Konstanz, Germany; COGENICS, Grenoble, France). For the transformation of *Y. lipolytica*, plasmid constructions were first linearized by digestion with *Not*I, generating expression cassettes displaying flanker zeta sequences that directed their introduction at the zeta docking platform present in the genome of *Y. lipolytica* JMY1212. The homologous mono-integration of the expression cassette at this platform is thus forced in this strain. Transformation was performed using the lithium acetate method [22] and Ura+ transformants were subsequently selected for on solid YNBcasaD medium (i.e. YNB containing 2 g/L casamino acids and 1 g/L glucose).

### 4. Production of free and anchored xylanase

#### 4.1. Production of recombinant TxXYN in *E. coli*

Mature TxXYN was produced in *E. coli* using JM109 (DE3) cells harboring the relevant pECXYL-R2 plasmid, according to Paes et O'Donohue [20]. 50 ml cultures in LB broth induced by IPTG 0.4 mM were performed in triplicate, and the overall xylanase activity was measured in the cell-free extract by the DNS method described in paragraph 5.

#### 4.2. Selection of a *Y. lipolytica* representative transformant

The screening of three individual transformants was performed as described elsewhere [16]. After centrifugation (5,000 g for 10 min), cells were washed twice using a physiological solution (9 g/L NaCl) and lyophilised. Supernatants were stored at 4°C for further analysis.

#### 4.3. Production of enzyme in optimal aeration conditions

25 mL of liquid medium (containing yeast extract, 10 g/L, bactotryptone, 20 g/L, and either oleic acid, 50 g/L, glucose, 100 g/L, or glycerol, 100 g/L, buffered with 100 mM phosphate, pH 6.8) contained in a baffled erlenmeyer flask (250 mL) were inoculated with a previously prepared culture grown overnight in YPD (yeast extract 10 g/L, bactopeptone 10 g/L, and glucose 10 g/L) medium, to obtain an initial cell density of OD_600_ = 0.6. The culture was incubated at 28°C with shaking (120 rpm) for 96 h (4 days) until the carbon source was completely consumed. HPLC was used to monitor the consumption of glucose and glycerol (column Aminex HPX-87H (300 mmx7,8 mm) equipped with a RI detector; oven temperature 50°C; mobile phase 0.5 mL/min H_2_SO_4_ 5 mM), while that of oleic acid was monitored visually by examining the turbidity of culture supernatants after centrifugation. At the end of the culture period, cells and culture supernatant were recovered and treated as described above.

### 5. Determination of xylanase activity

Xylanase activity was determined over a 10-minute period by monitoring birchwood xylan (BX) hydrolysis (5 g/L in 50 mM sodium acetate buffer pH 5.6) at 60°C using the 3,5 dinitrosalicylic acid (DNS) method [23]. This method quantifies the amount of soluble reducing sugars using a spectrophotometric analysis at 545 nm. In the case of supernatants, 50 µL of enzyme sample were added to 450 µL of BX suspension, whereas when working with cells, 10 mg (dry weight) were added to 1 mL BX suspension. One unit of xylanase activity (1 U) is defined as the quantity of xylanase needed to release 1 µmol of equivalent xylose per minute.

### 6. SDS-PAGE and Western Blot analysis

Crude supernatant was concentrated 10-fold using an Amicon Ultra-4 Centrifugal Filter Unit with 10 kDa cut-off (Merk Millipore, Bedford, MA, USA) and deglycosylated using endoglycosidase H (New England Biolabs, Beverly, MA, USA), according to manufacturer's instructions. Proteins were then analysed on Any kD Tris-Glycine SDS-PAGE (Biorad, Marnes-La-Coquette, France). After migration, the gel was submitted to Coomassie blue staining or further analysed by Western Blot.

For western blotting, the separated proteins were transferred from the gel to polyvinylidene difluoride (PVDF) membrane (Merk Millipore, Bedford, MA, USA) using a Biorad unit with transfer buffer (25 mM Tris pH 8.3, 192 mM glycine, 20% EtOH, 0.05% SDS). The membrane was subsequently blocked 45 minutes in TBS (Tris 50 mM pH 7.4, 150 mM NaCl), supplemented with 5% skimmed milk. The membrane was then incubated at 4°C overnight in TBS, supplemented with 5% w/v skimmed milk and primary mouse non position-specific His-Tag antibody 1∶2500 (THE from Genscript, Piscataway, NJ, US). The membrane was washed 3 times with TBS supplemented with 0.2% v/v Tween20 and incubated 1 h at room temperature in TBS supplemented with 5% w/v skimmed milk and secondary alkaline phosphatase-conjugated goat anti-mouse IgG antibody. The gel was washed again 3 times with TBS supplemented with 0.2% v/v Tween20. For detection, the PVDF membrane was incubated with a mixture of nitro blue tetrazolium chloride and 5-Bromo-4-chloro-3-indolyl phosphate.

## Results and Discussion

### 1. Production of free xylanase in *Y. lipolytica*

**Figure 1:**
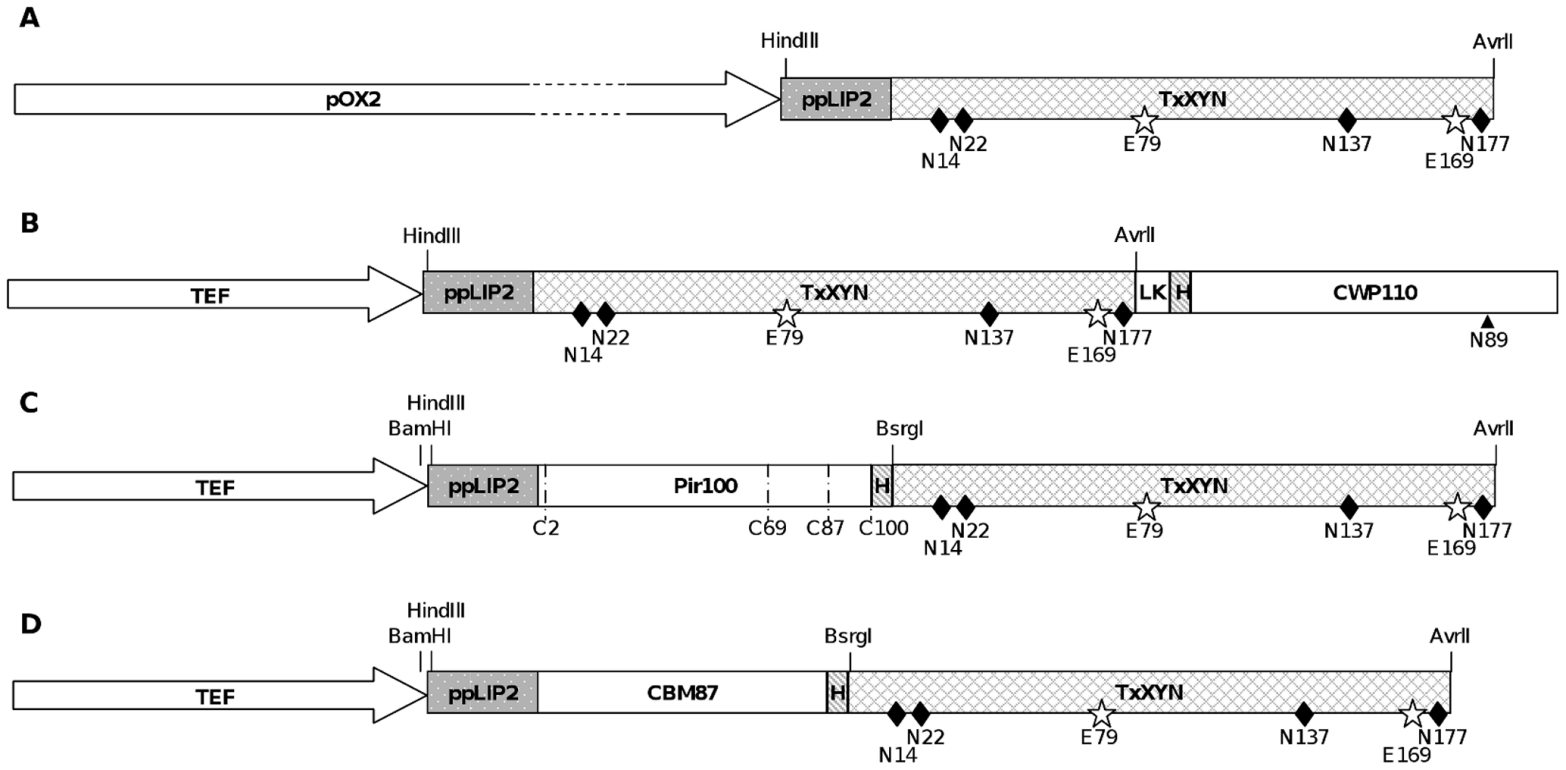
Schematic representation of the constructions used for production of free and anchored xylanase in *Y. lipolytica*. Constructions used for production of free TxXYN A) TxXYN fused with YlCWP110 C-terminal amino acids B), YlPir 100 C-terminal amino acids C) and YlCBM 87 C-terminal amino acids D). ppLIP2, preproLIP2 used as secretion signal peptide; H, 6 histidines tag; LK, 10 amino acids linker peptide; relevant restriction sites in the encoding genes are indicated above the resulting proteins, while relevant amino acids are indicated below (diamonds for the 4 N-Glycosylation sites and stars for the 2 catalytic glutamic acids in TxXYN, 4 cysteines in Pir100 and the asparagine as acceptor of the GPI anchor in CWP110).

Profiting from *Y. lipolytica*'s natural ability to secrete proteins, the LIP2 prepro sequence was fused to the sequence encoding TxXYN, creating the plasmid JMP61-POX2-*ppLIP2*-*TxXYN* (Figure 1A). The strategy proposed by Cambon *et al*. was utilized to select a transformant representative of the global population: three randomly selected transformants were cultivated in the medium Y_1_T_2_O_1_ [16]. Accordingly, the transformants achieved an average production of xylanase activity of ∼5,700 U/L (13.3% standard deviation), which is approximately 30% higher than the yield of TxXYN obtained when using *E. coli* as the expression host (4,452±381 U/L). Advantageously, the xylanase activity produced in *Y. lipolytica* was recovered from the culture supernatant, contrarily to production in *E. coli* which involves recovery from the cytosolic fraction, thus facilitating the subsequent protein recovery and purification steps.

### 2. Construction of fusion proteins

To compare three different cell wall anchoring systems in *Y. lipolytica*, the sequence encoding TxXYN was fused to three different protein anchors. All of the fusion proteins shared in common the *Y. lipolytica* Lip2 signal peptide and prosequence (ppLIP2, 33 amino acids) that addresses proteins to the extracellular medium [24].

The first of the fusion proteins contained the *Y. lipolytica* YlCWP1 Cell Wall Protein (accession number Q8TFK5, gene YALI0E18788g), which presents 28% overall identity with CWP1 from *S. cerevisiae* [25]. Y1CWP1 harbours a putative GPI-attachment site (Glycosyl-Phosphatidyl Inositol) that corresponds to Asn200. According to the literature, the GPI anchor is a post-translational modification composed of a postglycophospholipid structure that is added to the carboxyl terminus (ω-site) of proteins after proteolytic cleavage of a C-terminal propeptide. The presence of this anchor enables the covalent linkage of proteins to cell-wall β-1,6-glycans [26]. Consequently, to anchor proteins to the cell wall, the anchoring domain has to be present at the C-terminus of target proteins [27]. Accordingly, in this work the C-terminal part of YlCWP1 (110 amino acids), containing the GPI anchor domain, was fused to TxXYN at its C-terminal extremity, a strategy that has already been successfully employed to display other proteins at the cell wall of *Y. lipolytica* [13], [17], [28], [29]. Since the resultant protein TxXYN-CWP110 (Figure 1B) contains a truncated form of YlCWP1, the putative GPI-attachment site is located at Asn89 of the CWP110 domain (corresponding to Asn292 in the mature fusion protein).

The second anchoring system is composed of the C-terminal part (100 amino acids) of the Pir protein from *Y. lipolytica*, YlPir (accession number Q9C1F8, YALI0B20306g, 47% overall identity with Pir4 from *S. cerevisiae*). According to previous knowledge, ScPir4 only displays one Pir motif that is responsible for the attachment of Pir proteins to cell wall β-1,3-glycans [3], [30]. Contrarily, the sequence of YlPir reveals the absence of a characteristic repetitive motif [31]. The comparison of the C-terminal sequence (100 amino acids) of YlPir and six homologues, four from *S. cerevisiae* and two from *Candida albicans*, revealed 46% sequence identity and 79% sequence similarity (or interchangeable amino acids) between the seven proteins. Four conserved cysteine residues, previously shown to be involved in disulfide bonds that mediate cell-wall attachment [30], were identified in YlPir at positions 189, 255, 273 and 286. The Cys residues all form part of the 100 amino acid domain that was fused to TxXYN, leading to the fusion protein Pir100-TxXYN (Figure 1C).

The third anchoring system is based on the use of a protein domain that closely resembles the chitin-binding motif (CBM) from the endochitinase CTS1 of *S. cerevisiae*, which has previously been used to display GFP and lipase from *Aspergillus oryzae* on the surface of *A. oryzae* [5]. Reportedly, this protein binds non-covalently to cell wall structural proteins [32]. In *Y. lipolytica*, we found a homolog of this protein, which is encoded by the gene YALI0D22396g. The C-terminal part (87 amino acids) of the YALI0D22396g-encoded protein presents 46% similarity (32% identity) with the CTS1 CBM. Therefore, we fused this C-terminal YlCBM domain to TxXYN, procuring the fusion protein CBM87-TxXYN (Figure 1D).

It is noteworthy that previously Pir and CBM were linked to target proteins at both the N and C-terminal extremities [3], [5]. In the case of the former, the position of Pir did not appear to be important with regard to cell wall anchoring. However, in the case of CBM, previous work on CBM/lipase fusions revealed that the presence of a cell-wall bound N-terminal fusion (i.e. CBM-lipase) was four-fold higher than the C-terminal fusion (i.e. lipase-CBM) [5]. Therefore, in the present study we decided to construct only N-terminal fusions, thus Pir100-TxXYN and CBM87-TxXYN. This decision was further supported by the fact that the 3D structure of TxXYN clearly shows that its N- and C-termini are spatially very close [33].

### 3. Production of anchored xylanase

#### 3.1. Selection of a representative transformant for each construction

**Figure 2:**
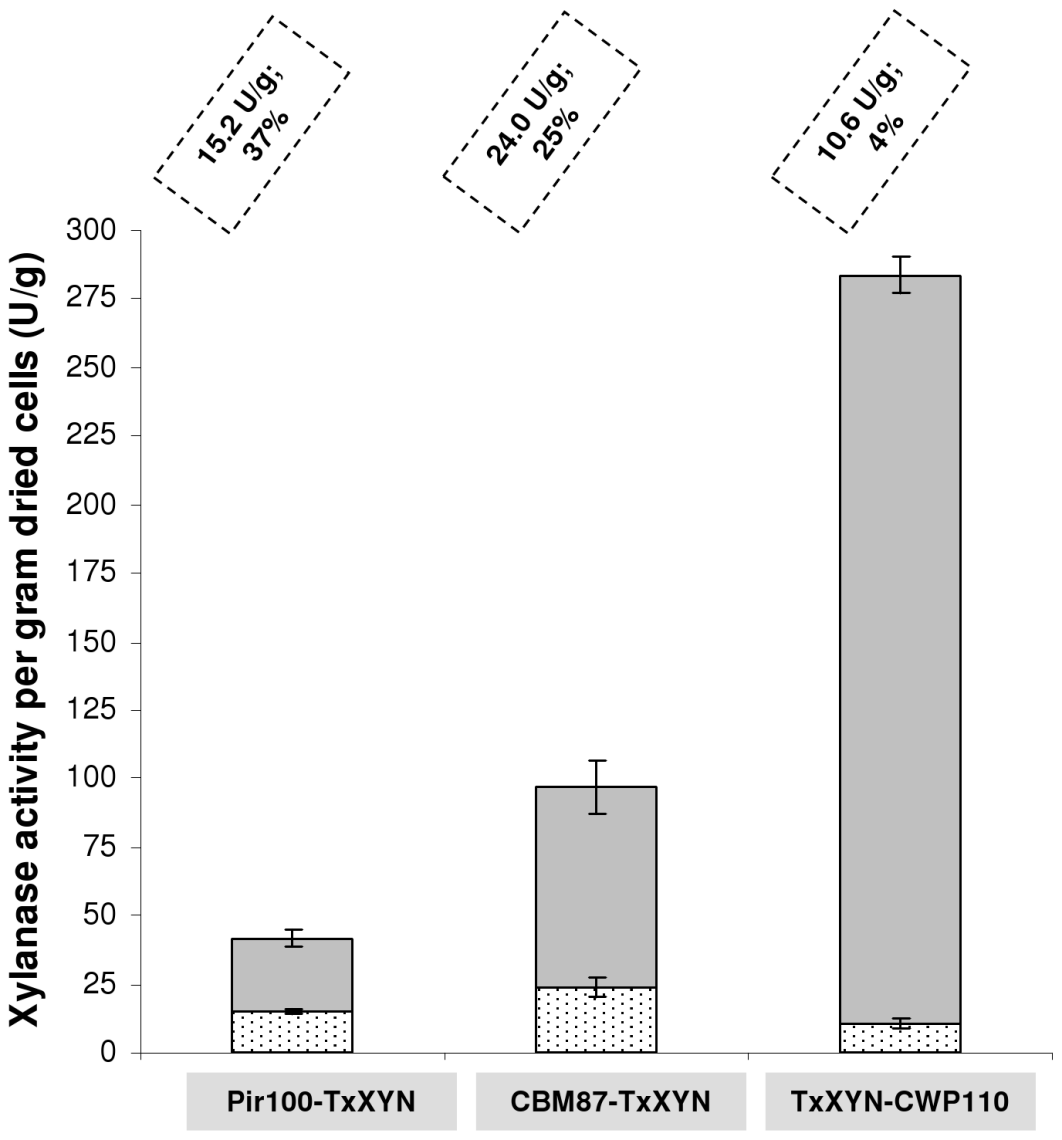
Xylanase activities as determined in cell walls (lyophilised cell pellets) and growth medium when producing the three fusion proteins in *Y. lipolytica* JMY1212. Xylanase activities determined for strain *Y. lipolytica* JMY1212 transformed with JMP62-TEF-ppLIP2-CBM87-TxXYN, JMP62-TEF-ppLIP2-Pir100-TxXYN, JMP62-TEF-ppLIP2-TxXYN-CWP110 and cultivated overnight in 10 g/L oleic acid. Xylanase units per gram dried cells in culture supernatant and lyophilised cell pellets are displayed in grey and spotted bars, respectively. Units of bound xylanase per gram dried cells and corresponding percentages are indicated for each construction. The % activity anchored on cells is the ratio between total units in cell pellet and total units in the whole culture (94 mg cell pellet and 10 mL supernatant). Mean and standard deviation of three experiments are presented.

As mentioned above, for each of the three fusion proteins, TxXYN-CWP110, Pir100-TxXYN and CBM87-TxXYN respectively, three individual *Y. lipolytica* transformants were randomly chosen for further analysis. Xylanase activity was assayed on the non-internalisable polysaccharide xylan in both culture supernatants and in lyophilized cell pellets, and the percentage of cell-bound xylanase was expressed as the ratio between cell-bound activity and that of the whole culture (Figure 2). No cell-bound xylanase activity was detected in the control strain JMY1212 (data not shown). The coefficients of variation (determined while expressing the standard deviation relative to the mean) obtained for xylanase activity in the supernatants and lyophilized cell pellets being less than 15%, the three transformants were considered to be genetically identical and thus one transformant representative of each construction was selected for further experiments.

The lowest amount of bound xylanase (10.6 U/g) was obtained when using the CWP anchoring domain. This only represented 4% of total activity, since most of the xylanase appeared to be present in the culture supernatant (2.6 U/mL corresponding to 273 U/g dried cells). The Pir100-TxXYN construction was better anchored to the cell wall, since the cell-bound activity reached 15.2 U/g, representing 37% of total activity. Finally, the CBM87-TxXYN fusion provided 24 U/g of cell-bound xylanase activity, indicating that the overall performance of this system was higher than the other two, although cell-bound xylanase activity only represented 25% of total activity.

The presence of xylanase activity in the culture supernatants, irrespective of the nature of the protein fusions, is no doubt the result of non-specific proteolysis. This phenomenon has already been observed when using the anchoring proteins described in this study. Moreover, previous work indicates that the performance of the anchoring system is very much partner protein dependent. Accordingly, when using CBM, Tabuchi *et al.* failed to detect any GFP in the supernatant of cells expressing CBM-GFP, but observed the significant presence in the culture supernatant of the lipase TglA, when this was produced as a CBM-fusion [5]. Similarly, previous studies, in which ScPir4 has been linked to *Bacillus sp.* proteins, lipase A or xylanase A for display in *S. cerevisiae*, revealed that while 100% of ScPir4-xylanase was cell-bound, only 40% of the lipase A appeared to be anchored to the cell wall [3], [4]. Moreover, when a ScPir4 variant was used, which lacks the aforementioned functionally important Cys residues, only 12% of either protein was found to be cell bound. Finally, when comparing the ability of different endogenous YlCWPs to display Lip2 lipase from *Y. lipolytica* on the surface of *Y. lipolytica*, Yuzbasheva *et al.* found that 30% of lipase activity was localized in the supernatant [13]. These authors suggested that this imperfect cell-wall anchoring could be due to either proteolysis and/or the poor accessibility of the GPI anchor. Overall, the results reported here are consistent with previous findings and appear to confirm that perfect cell-wall anchoring of proteins is quite difficult to achieve, possibly due to host proteolytic activity, which is discussed later.

#### 3.2. Optimisation of the enzyme expression

**Figure 3:**
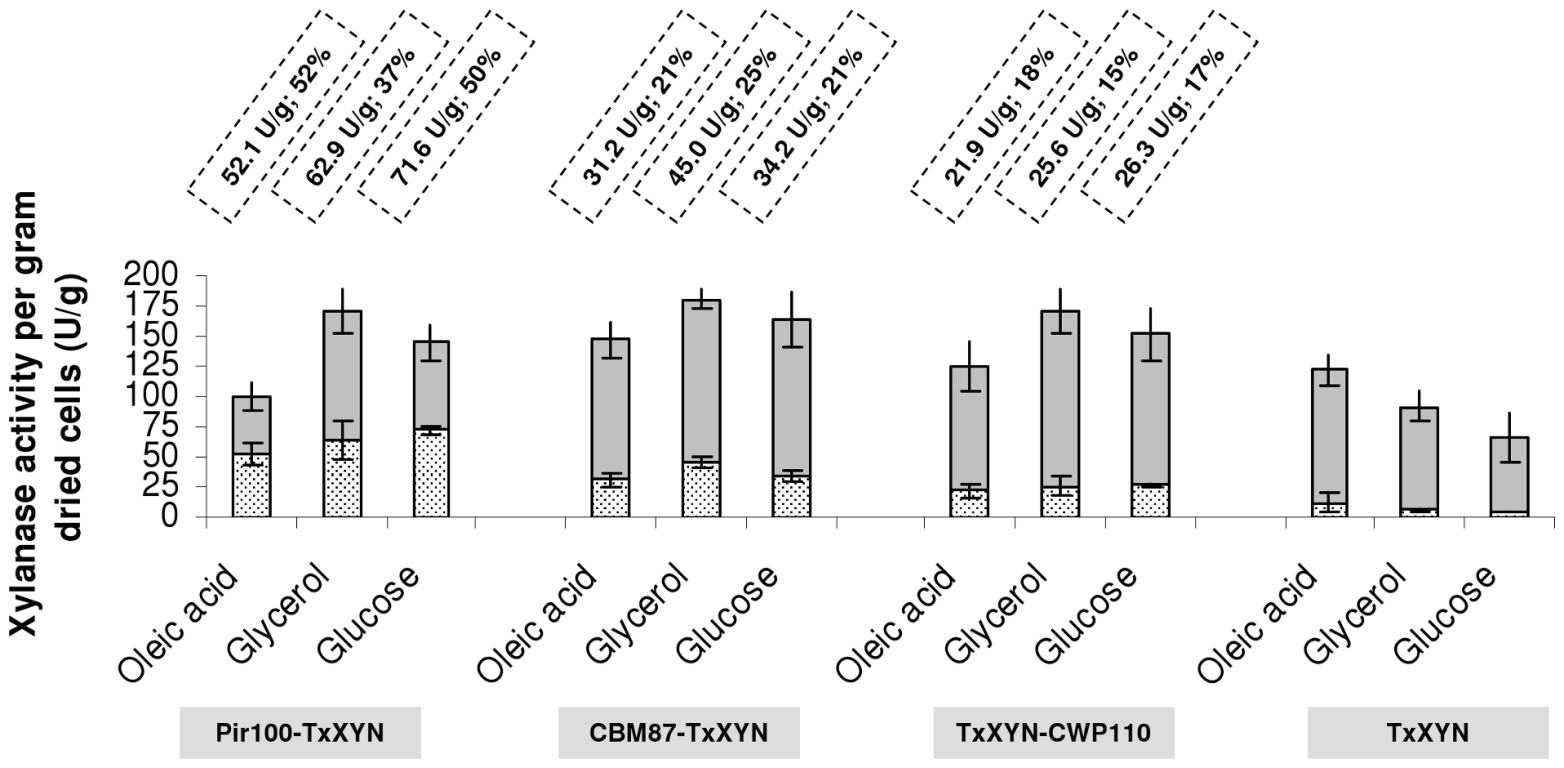
Immobilisation efficiencies of different anchoring systems depending on the carbon source. Total xylanase activities obtained for strain *Y. lipolytica* JMY1212 transformed with JMP62-TEF-ppLIP2-Pir100-TxXYN, JMP62-TEF-ppLIP2-CBM87-TxXYN, JMP62-TEF-ppLIP2-TxXYN-CWP110 cultivated 4 days with 3 different carbon sources; strain JMY1212 transformed with JMP61-POX2-ppLIP2-TxXYN was used as control. Xylanase units per gram dried cells in culture supernatant (25 mL) and lyophilised cell pellets (0.7, 0.89 and 1 g in glycerol, glucose and oleic acid, respectively) are displayed in grey and spotted bars, respectively. Units of bound xylanase per gram dried cells and corresponding percentages are indicated for each construction. Mean and standard deviation of four experiments are presented.

In an attempt to optimize protein expression the different transformants were cultured in 25 mL of medium over a longer period in larger volume (250 mL baffled rlenmeyer flasks), thus maximizing aeration. For all fusion proteins, the activity in the supernatant is released since the beginning of the expression. The distribution of anchored versus secreted protein was followed over 7 days. It remained identical until the beginning of the stationary phase and then decreased. Cultures were consequently stopped when carbon source depletion occurred at the beginning of the stationary phase, which corresponds to a 4-day cultivation. Since little is known about the impact of carbon source on the production and regulation of cell wall proteins in *Y. lipolytica*, especially endogenous YlCWP, YlPir and YlCBM, this parameter was studied, while keeping the amount of carbon constant (39.1±0.9 g/L) in each case (Figure 3). Accordingly, the strain designed to secrete TxXYN showed the highest xylanase activity (109 U/g dried cells in supernatant) when oleic acid was used as the carbon source. This is unsurprising, since the activity of the POX2 promoter is induced by fatty acids. Interestingly, even in this strain, some xylanase activity (7% of the total activity) was cell-bound, probably ongoing the secretion pathway. Likewise, when glycerol or glucose was used as carbon source, the proportion of cell-bound (trapped) xylanase proportion was very similar (5 and 6% respectively).

After a 96-h culture period and total consumption of the carbon source, the dry cell weights were on average 28 g/L (glycerol), 35 g/L (glucose) and 40 g/L (oleic acid). The total amount of xylanase produced per gram dried cells (secreted activity in supernatant plus cell-bound activity) ranged from 100 U/g (Pir100-TxXYN cultivated in oleic acid) to 180 U/g (CBM87-TxXYN cultivated in glycerol), with a percentage of cell-bound xylanase ranging from 15% (TxXYN-CWP110 cultivated in glycerol) to 52% (Pir100-TxXYN cultivated in oleic acid). The highest activity was observed in glycerol (174±6 total U/g compared to 153±10 total U/g in glucose and 124±23 total U/g in oleic acid total U/g). Total xylanase production on both glucose and glycerol is rather independent of the nature of the protein anchor system employed, whereas it differs significantly in oleic acid. These results imply that protein expression might be the limiting step and, as a consequence, that a way to increase the level of cell-bound xylanase would be to employ multiple gene copies and/or use a stronger promoter.

As revealed in the preliminary experiments performed in 10 mL medium, when oleic acid was used as the carbon source in larger culture volumes, the TxXYN-CWP110 construction still provided the lowest amount of cell-anchored xylanase (21.9 U/g), although this represents a 107% improvement on the small-scale experiment. The CBM87-TxXYN construction was better (31.2 U/g, 29% improvement) and Pir100-TxXYN provided the best results (52.1 U/g, 242% improvement). Regarding the CBM system, it is possible that accessibility to chitin is limited in yeast and thus the amount of expressed CBM87-TxXYN saturated the available chitin binding sites. Such phenomenon has already been observed in the case of the CWP anchor system. Strains deleted for SED1, a major cell wall protein, enabled protein binding to be increased, by reducing competition for the cell surface [34]. Overall, our results revealed that Pir100-TxXYN is the most efficient system, both in terms of the quantity of cell-bound xylanase activity (52 U/g) and of the percentage of cell-bound activity (52%) versus total activity.

Apparently, irrespective of the anchoring system employed, the carbon source (glucose, oleic acid and glycerol) had no significant effect either on the amount of anchored protein or on the unbound versus cell-bound protein ratio. Moreover, the partition between bound and unbound enzyme is principally a function of the anchor system. Indeed, free xylanase levels ranged from 48 to 85% for Pir100-TxXYN and TxXYN-CWP110 respectively.

In summary, Pir100-TxXYN was found to be the best system for the anchoring of TxXYN onto *Y. lipolytica* cells, achieving a maximum of 71.6 U/g dry cells when cultured in glucose, with only 3.4 U/mL being found in the growth medium (i.e. 52% is cell-bound). To our knowledge, this represents the most efficient cell-surface anchoring system for xylanases described to date, since the next best result (49 U/g) is that of the *Cellulomonas fimi* xylanase displayed on *E. coli* cells [35]. Therefore, *Y. lipolytica* Pir100-TxXYN could well be a useful whole cell biocatalyst. Nevertheless, one may not assume that this result can be generalized to all enzymes as it is observed with conventional immobilization techniques. Indeed, the superiority of this system with TxXYN could be due either to a larger amount of protein anchored on the cell wall or on the higher accessibility of the Pir-TxXYN fusion to large substrate.

#### 3.3. Characterization of the activity leak phenomenon

In order to better understand the leaching of xylanase from the cell wall, western blotting was employed to monitor proteins in the culture supernatant. To avoid any interference by protein glycosylation (TxXYN displays 4 potential N-glycosylation sites) (Figure 1), the free protein fraction present in the supernatant was first deglycosylated using endoglycosidase H and two different anti-Histidine antibodies were employed. The first is an anti-His antibody that only reacts with C-terminal His-tag, while the second one recognizes C-terminal, N-terminal, and internal His tagged fusion proteins.

**Figure 4:**
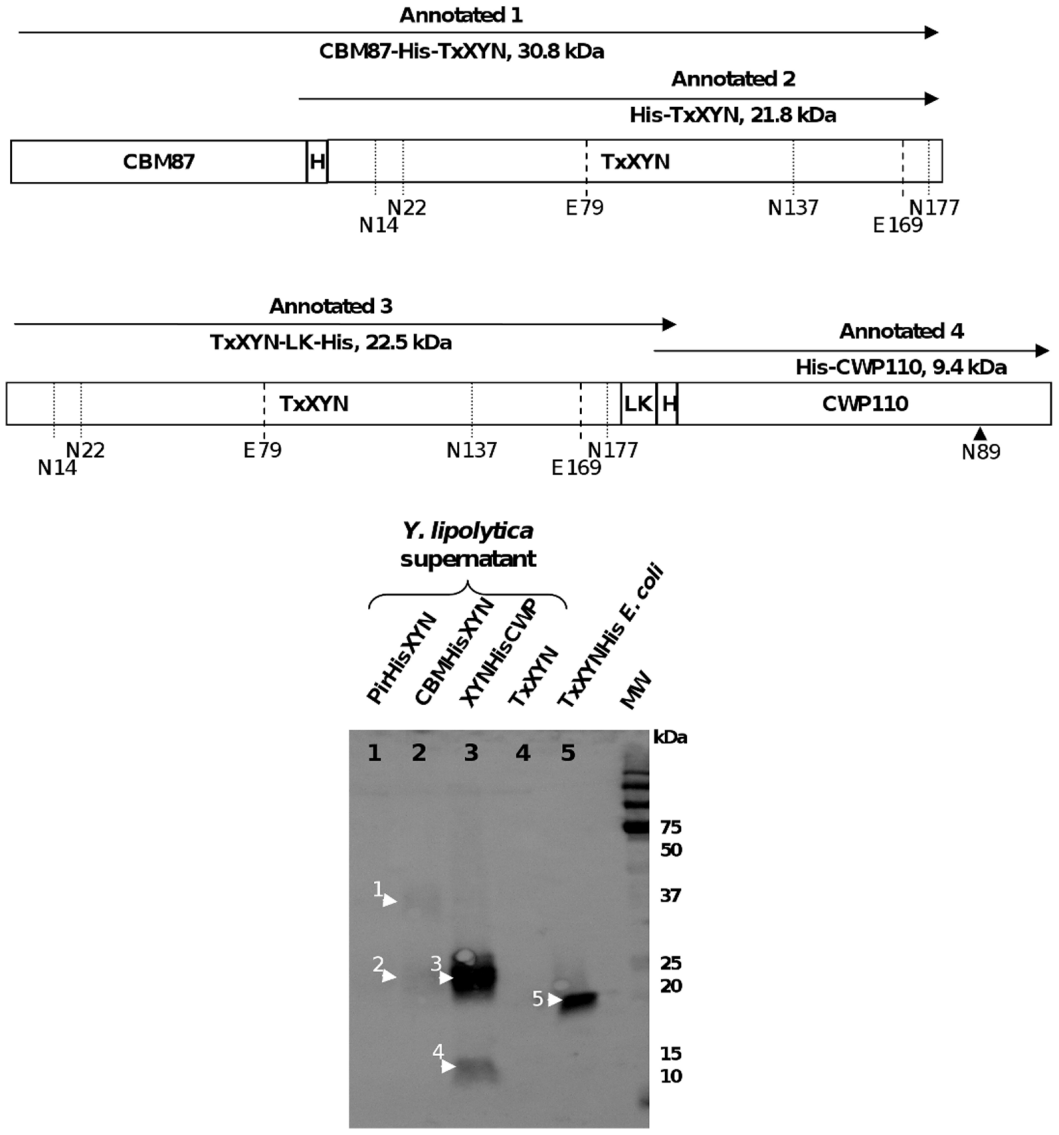
Western blot analysis of xylanase fusion proteins found in supernatant fractions. Proteins released in supernatant were revealed with non position-specific antiHis primary antibodies. Supernatants were concentrated 10 times and deglycosylated with endoglycosidase H prior to western blot analysis. A schematic representation of proteins detected in SDS-PAGE analysis is added.

As presented in Figure 4, a strong signal was observed at a position corresponding to a free protein species of approximately 23 kDa in the supernatant arising from the strain *Y. lipolytica* TxXYN-CWP110 (lane 3, annotated 3). This species was also revealed by the C-terminal specific anti-His antibody (data not shown). Importantly, the estimated molecular weight of this protein species is slightly higher than that of C-terminal His-tagged TxXYN produced by *E. coli* (theoretical mass 21.8 kDa, lane 5 annotated 5). This difference is consistent with the presence of the linker, LK (0.7 kDa), and thus strongly implies that this protein species is the result of the degradation of TxXYN-CWP110 into TxXYN-LK-His (theoretical mass 22.5 kDa). One might speculate that this degradation could be favoured by the presence of an unstructured zone between the 2 fused proteins, which would be good target for proteolysis. Moreover, the non position-specific anti-His antibody revealed a second free protein having an estimated Mw of 12 kDa (lane 3, annotated 4). This protein could well be the other part of the initial cell-bound protein fusion (i.e. His-CWP110), whose theoretical Mw is 9.4 kDa. The difference in molecular weight (i.e. 2.6 kDa) could be due to O-glycosylation (often mentioned for cell wall proteins) that would not be affected by endoglycosidase H treatment.

In the supernatant arising from the strain *Y. lipolytica* CBM87-TxXYN (Figure 4, lane 2), a protein migrating to a position consistent with a Mw of 22 kDa was revealed using the non position-specific antibody, but not the C-terminal specific anti-His antibody (lane 2, annotated 2). This species most likely corresponds to His-TxXYN (theoretical Mw of 21.8 kDa) and corroborates the hypothesis that protein degradation occurs between the 2 fused proteins. A second high molecular weight protein species (approximately 37 kDa, lane 2, annotated 1) was also detected in the supernatant using the non position-specific antibody. This is probably the result of leaching of the intact protein fusion CBM87-TxXYN, which displays a theoretical Mw of 30.8 kDa. Once again, the difference between the observed and theoretical molecular weights could be the result of O-glycosylation.

Finally, no protein appeared in the supernatant of *Y. lipolytica* Pir100-TxXYN (Figure 4, lane 1), which is consistent with the low activity measured in this supernatant and the fact that over half of the protein is cell-bound.

Overall, the results presented above confirm that proteolysis is responsible for the leaching of cell-anchored TxXYN into the culture medium. Moreover, the data strongly suggest that the region that joins the xylanase to the anchor protein is particularly prone to proteolysis. Interestingly, the above data also reveal that in the case of CBM87 protein fusion, intact CBM87-TxXYN can be found in the supernatant, which is consistent with the idea that the cell surface could become saturated with cell wall proteins when these are too strongly expressed. This suggests that the immobilisation process could be further improved either by using a combination of different kinds of cell wall proteins for displaying the same biocatalyst of interest or by deleting host endogenous cell wall proteins in order to liberate some anchoring sites.

#### 3.4. Activity of TxXYN in *Y. lipolytica* culture conditions

The usefulness of the xylanase-displaying *Y. lipolytica* as a whole-cell biocatalyst depends on the ability of TxXYN to perform hydrolysis of arabinoxylans in *Y. lipolytica* growth conditions. Regarding pH, TxXYN retains 60% of its activity over the range pH 4 to 8, and its optimal activity is obtained at pH 6 [33]. The optimal pH for *Y. lipolytica* growth being 5.6, it was chosen for the following experiments. Regarding temperature, TxXYN is reported to be optimally active at 60°C, whereas the optimal temperature for *Y. lipolytica* growth is 28°C. Cells bearing Pir-TxXYN retained 33% activity at 28°C relative to activity at 60°C. In addition, the remaining activity at 28°C was found to be 40% for the Pir-TxXYN suspension (cells + supernatant, which corresponds to the total available xylanase in the context of a continuous process). This corresponds to a classic activity decrease in agreement with Arrhenius law.

## Conclusion

Using *Y. lipolytica* JMY1212 and a bacterial xylanase, we have accurately compared three cell wall anchoring systems for protein display. Likewise, it has been possible to demonstrate that YlPir is an efficient protein anchor for the xylanase producing yeast that display high cell-bound xylanase activity and which constitutes the best whole cell xylanase display system so far described in the literature. Importantly, YlPir is three-fold more efficient than YlCWP, which is the only other display system that has so far been described for *Y. lipolytica*. Our results indicate that even higher cell-bound xylanase activity might be obtained by deleting endogenous YlPir, although this remains to be tested.

Overall, this work constitutes the first step in the development of a biotechnologically-relevant yeast that will be used for the hydrolysis of arabinoxylan-containing biomass.

## References

[1] Sergeeva A, Kolonin MG, Molldrem JJ, Pasqualini R, Arap W (2006) Display technologies: application for the discovery of drug and gene delivery agents. Adv Drug Deliv Rev 58: 1622–1654. 17123658 10.1016/j.addr.2006.09.018 PMC1847402

[2] Breinig F, Schmitt MJ (2002) Spacer-elongated cell wall fusion proteins improve cell surface expression in the yeast *Saccharomyces cerevisiae* . Appl Microbiol Biotechnol 58: 637–644. 11956747 10.1007/s00253-002-0939-2

[3] Andres I, Gallardo O, Parascandola P, Javier Pastor FI, Zueco J (2005) Use of the cell wall protein Pir4 as a fusion partner for the expression of *Bacillus* sp. BP-7 xylanase A in *Saccharomyces cerevisiae* . Biotechnol Bioeng 89: 690–697. 15685601 10.1002/bit.20375

[4] Mormeneo M, Andres I, Bofill C, Diaz P, Zueco J (2008) Efficient secretion of *Bacillus subtilis* lipase A in *Saccharomyces cerevisiae* by translational fusion to the Pir4 cell wall protein. Appl Microbiol Biotechnol 80: 437–445. 18626643 10.1007/s00253-008-1549-4

[5] Tabuchi S, Ito J, Adachi T, Ishida H, Hata Y, et al (2010) Display of both N- and C-terminal target fusion proteins on the *Aspergillus oryzae* cell surface using a chitin-binding module. Appl Microbiol Biotechnol 87: 1783–1789. 20499230 10.1007/s00253-010-2664-6 PMC2903697

[6] Liu W, Zhao H, Jia B, Xu L, Yan Y (2009) Surface display of active lipase in *Saccharomyces cerevisiae* using Cwp2 as an anchor protein. Biotechnol Lett 32: 255–260. 19821073 10.1007/s10529-009-0138-7

[7] Shiraga S, Kawakami M, Ishiguro M, Ueda M (2005) Enhanced reactivity of *Rhizopus oryzae* lipase displayed on yeast cell surfaces in organic solvents: potential as a whole-cell biocatalyst in organic solvents. Appl Environ Microbiol 71: 4335–4338. 16085821 10.1128/AEM.71.8.4335-4338.2005 PMC1183351

[8] Murai T, Ueda M, Shibasaki Y, Kamasawa N, Osumi M, et al (1999) Development of an arming yeast strain for efficient utilization of starch by co-display of sequential amylolytic enzymes on the cell surface. Appl Microbiol Biotechnol 51: 65–70. 10077821 10.1007/s002530051364

[9] Murai T, Ueda M, Yamamura M, Atomi H, Shibasaki Y, et al (1997) Construction of a starch-utilizing yeast by cell surface engineering. Appl Environ Microbiol 63: 1362–1366. 9097432 10.1128/aem.63.4.1362-1366.1997 PMC168429

[10] Sakamoto T, Hasunuma T, Hori Y, Yamada R, Kondo A (2012) Direct ethanol production from hemicellulosic materials of rice straw by use of an engineered yeast strain codisplaying three types of hemicellulolytic enzymes on the surface of xylose-utilizing *Saccharomyces cerevisiae* cells. J Biotechnol 158: 203–210. 21741417 10.1016/j.jbiotec.2011.06.025

[11] Yanase S, Yamada R, Kaneko S, Noda H, Hasunuma T, et al (2010) Ethanol production from cellulosic materials using cellulase-expressing yeast. Biotechnol J 5: 449–455. 20349451 10.1002/biot.200900291

[12] Murai T, Ueda M, Atomi H, Shibasaki Y, Kamasawa N, et al (1997) Genetic immobilization of cellulase on the cell surface of *Saccharomyces cerevisiae* . Appl Microbiol Biotechnol 48: 499–503. 9390459 10.1007/s002530051086

[13] Yuzbasheva EY, Yuzbashev TV, Laptev IA, Konstantinova TK, Sineoky SP (2011) Efficient cell surface display of Lip2 lipase using C-domains of glycosylphosphatidylinositol-anchored cell wall proteins of *Yarrowia lipolytica* . Appl Microbiol Biotechnol 91: 645–654. 21494864 10.1007/s00253-011-3265-8

[14] Bordes F, Fudalej F, Dossat V, Nicaud JM, Marty A (2007) A new recombinant protein expression system for high-throughput screening in the yeast *Yarrowia lipolytica* . J Microbiol Methods 70: 493–502. 17669530 10.1016/j.mimet.2007.06.008

[15] Nicaud JM, Madzak C, van den Broek P, Gysler C, Duboc P, et al (2002) Protein expression and secretion in the yeast *Yarrowia lipolytica* . FEMS Yeast Res 2: 371–379. 12702287 10.1016/S1567-1356(02)00082-X

[16] Cambon E, Piamtongkam R, Bordes F, Duquesne S, Laguerre S, et al (2010) A new *Yarrowia lipolytica* expression system: An efficient tool for rapid and reliable kinetic analysis of improved enzymes. Enzyme and Microbial Technology 47: 91–96.

[17] Yu XJ, Madzak C, Li HJ, Chi ZM, Li J (2010) Surface display of acid protease on the cells of *Yarrowia lipolytica* for milk clotting. Appl Microbiol Biotechnol 87: 669–677. 20349181 10.1007/s00253-010-2549-8

[18] Dumon C, Song L, Bozonnet S, Fauré R, O'Donohue MJ (2011) Progress and future prospects for pentose-specific biocatalysts in biorefining. Process Biochemistry 47: 1359–5113.

[19] Beg QK, Kapoor M, Mahajan L, Hoondal GS (2001) Microbial xylanases and their industrial applications: a review. Appl Microbiol Biotechnol 56: 326–338. 11548999 10.1007/s002530100704

[20] Paes G, O'Donohue MJ (2006) Engineering increased thermostability in the thermostable GH-11 xylanase from *Thermobacillus xylanilyticus* . J Biotechnol 125: 338–350. 16644050 10.1016/j.jbiotec.2006.03.025

[21] Querol A, Barrio E, Huerta T, Ramon D (1992) Molecular monitoring of wine fermentations conducted by active dry yeast strains. Appl Environ Microbiol 58: 2948–2953. 16348768 10.1128/aem.58.9.2948-2953.1992 PMC183031

[22] Duquesne S, Bordes F, Fudalej F, Nicaud JM, Marty A (2012) The yeast *Yarrowia lipolytica* as a generic tool for molecular evolution of enzymes. Methods Mol Biol 861: 301–312. 22426726 10.1007/978-1-61779-600-5_18

[23] Miller GL (1959) Use of dinitrosalicylic acid reagent for determination of reducing sugar. Anal Chem 31: 426.

[24] Pignede G, Wang HJ, Fudalej F, Gaillardin C, Seman M, et al (2000) Characterization of an extracellular lipase encoded by LIP2 in *Yarrowia lipolytica* . J Bacteriol 182: 2802–2810. 10781549 10.1128/jb.182.10.2802-2810.2000 PMC101989

[25] Jaafar L, Zueco J (2004) Characterization of a glycosylphosphatidylinositol-bound cell-wall protein (GPI-CWP) in *Yarrowia lipolytica* . Microbiology 150: 53–60. 14702397 10.1099/mic.0.26430-0

[26] Paulick MG, Bertozzi CR (2008) The glycosylphosphatidylinositol anchor: a complex membrane-anchoring structure for proteins. Biochemistry 47: 6991–7000. 18557633 10.1021/bi8006324 PMC2663890

[27] Gerber LD, Kodukula K, Udenfriend S (1992) Phosphatidylinositol glycan (PI-G) anchored membrane proteins. Amino acid requirements adjacent to the site of cleavage and PI-G attachment in the COOH-terminal signal peptide. J Biol Chem 267: 12168–12173. 1601882

[28] Ni X, Yue L, Chi Z, Li J, Wang X, et al (2009) Alkaline protease gene cloning from the marine yeast *Aureobasidium pullulans* HN2-3 and the protease surface display on *Yarrowia lipolytica* for bioactive peptide production. Mar Biotechnol (NY) 11: 81–89. 18629587 10.1007/s10126-008-9122-9

[29] Yue L, Chi Z, Wang L, Liu J, Madzak C, et al (2008) Construction of a new plasmid for surface display on cells of *Yarrowia lipolytica* . J Microbiol Methods 72: 116–123. 18155308 10.1016/j.mimet.2007.11.011

[30] Castillo L, Martinez AI, Garcera A, Elorza MV, Valentin E, et al (2003) Functional analysis of the cysteine residues and the repetitive sequence of *Saccharomyces cerevisiae* Pir4/Cis3: the repetitive sequence is needed for binding to the cell wall beta-1,3-glucan. Yeast 20: 973–983. 12898712 10.1002/yea.1016

[31] Jaafar L, Moukadiri I, Zueco J (2003) Characterization of a disulphide-bound Pir-cell wall protein (Pir-CWP) of *Yarrowia lipolytica* . Yeast 20: 417–426. 12673625 10.1002/yea.973

[32] Cappellaro C, Mrsa V, Tanner W (1998) New potential cell wall glucanases of *Saccharomyces cerevisiae* and their involvement in mating. J Bacteriol 180: 5030–5037. 9748433 10.1128/jb.180.19.5030-5037.1998 PMC107536

[33] Harris GW, Pickersgill RW, Connerton I, Debeire P, Touzel JP, et al (1997) Structural basis of the properties of an industrially relevant thermophilic xylanase. Proteins 29: 77–86. 9294868

[34] Kuroda K, Matsui K, Higuchi S, Kotaka A, Sahara H, et al (2009) Enhancement of display efficiency in yeast display system by vector engineering and gene disruption. Appl Microbiol Biotechnol 82: 713–719. 19123001 10.1007/s00253-008-1808-4

[35] Chen YP, Hwang IE, Lin CJ, Wang HJ, Tseng CP (2012) Enhancing the stability of xylanase from *Cellulomonas fimi* by cell-surface display on *Escherichia coli* . J Appl Microbiol 112: 455–463. 22226305 10.1111/j.1365-2672.2012.05232.x

